# Intra-Individual Double Burden of Overweight and Micronutrient Deficiencies among Vietnamese Women

**DOI:** 10.1371/journal.pone.0110499

**Published:** 2014-10-15

**Authors:** Arnaud Laillou, Elizabeth Yakes, Thi Hop Le, Frank Tammo Wieringa, Bach Mai Le, Regina Moench-Pfanner, Jacques Berger

**Affiliations:** 1 UNICEF, Maternal Child Health and Nutrition section, Phnom Penh, Cambodia; 2 University of New Mexico, Department of Individual, Family and Community Education and Department of Family and Community Medicine, Albuquerque, New Mexico, United States of America; 3 National Institute of Nutrition (NIN), Nutrition section, Hanoi, Vietnam; 4 Institut de Recherche pour le Développement (IRD), Unité Mixte de Recherche 204 “Prévention des malnutritions et des pathologies associées”, Montpellier, France; 5 Global Alliance for Improved Nutrition (GAIN), Singapore Office, Singapore, Singapore; Faculty of Biology, Spain

## Abstract

**Background:**

Vietnamese Living Standard Surveys showed that the rate of overweight and obese in Vietnamese adults doubled between 1992 and 2002, from 2% to 5.5%, respectively with no significant difference in the proportions of overweight/obesity between men and women.

**Objectives:**

Considering the increasing public health concern over the double burden of malnutrition in Vietnam, we investigated micronutrient deficiencies among women of reproductive age according to their Body Mass Index.

**Methods:**

A transversal study was conducted in 2010 among 1530 women of reproductive age from 19 provinces. Participating women were asked to give a non-fasting blood sample for plasma iron, vitamin A, folate, vitamin B_12_ and zinc assessment.

**Results:**

Although % body fat was associated with haemoglobin, ferritin, retinol and zinc concentrations, BMI category was only associated with marginal vitamin A status (19% among underweight vs 7% among overweight/obese; p<0.0001) and not with iron deficiency anemia, zinc deficiency, vitamin B_12_ deficiency or folate status. The prevalence of iron, and vitamin B_12_ deficiencies was respectively 11.4% and 15% among the 20% overweight/obese women; prevalence of zinc deficiency and marginal/deficient folate status was much higher, affecting respectively 61.1% and 25.8%. Intra-individual double burden of malnutrition (overweight/obesity (OW) and micronutrient deficiency) was observed among 2.0% for OW-anemia, 2.3% OW-iron deficient, 3.0% for OW-Vitamin B_12_ deficiency, 12.2% for OW-Zinc deficiency and 5.2% for OW-marginal/deficient folate status.

**Conclusions:**

This large, cross-sectional survey demonstrated that micronutrient deficiencies are an issue across the weight spectrum among women in Vietnam, with only vitamin A status being better among overweight than underweight women. It is therefore essential for Vietnam to actively prevent women of reproductive age from overweight/obesity and at same time to control micronutrient deficiencies in this population to limit their economic and health consequences.

## Background

Obesity is a major health concern worldwide. According to the World Health Organization (WHO), there were approximately 1.5 billion overweight adults and 300 million obese women worldwide in 2008 [1]. Rapid social change, urbanisation and changes in the food supply and dietary patterns, along with demographic and other behaviour changes has caused a “nutrition transition” in many lower- and middle income countries. This transition has been characterized by an increase in overweight/obesity and related chronic diseases [2]. For example, economic growth, ageing of populations and urbanization with the associated lifestyle changes is likely to lead to a 54% increase in worldwide diabetes prevalence by 2030 [3].

Cardiovascular diseases and their risk factors, such as hypertension and obesity, are emerging as the leading causes of morbidity and mortality in many developing countries, including Vietnam [4]. As with many countries experiencing rapid economic growth, Vietnam is facing the so-called “double burden of malnutrition,” defined as the presence of both under-nutrition and over-nutrition in the population[5]. The Vietnamese Living Standard Surveys showed that the prevalence of overweight(BMI≥25 kg/m^2^) in Vietnamese adults doubled between 1992 and 2002, from 2% to 5.5%, respectively [6]. The National Adult Obesity study, using the Asian BMI cut-off of 23 kg/m^2,^_,_ showed an even higher prevalence of overweight in 2005 (16.3%), a sharp increase compared to 11.7% in 2000 [5]. This situation is likely to worsen, as a recent survey in Ho Chi Minh showed that the prevalence of combined overweight/obesity increased from 13.8% to 21.0% in a 6-year period among adolescents [7].

Individuals experiencing over-nutrition (overweight/obesity) due to excess energy intake may also suffer from micronutrient deficiencies. This has been particularly showed in relation to iron deficiency [8]. In addition, some recent studies showed that inadequate vitamin D and/or zinc status have been associated overweight and obesity and other metabolic syndromes [9]–[11]. Given the health risks associated with both excess adiposity and micronutrient deficiencies, their co-occurrence may have particularly dire consequences for the health of a population. The recent 2010 Vietnamese nationwide micronutrient deficiencies study showed high prevalence of zinc deficiency (∼67%) and vitamin B_12_ deficiency (∼12%) among women of reproductive age [12]. Prevalence of folate deficiency and vitamin A deficiency were considered negligible, but approximately 25% of women had marginal folate status and low iron stores, while 14% had marginal vitamin A status [12]. This survey also found an overweight/obesity (BMI≥23 kg/m^2^) prevalence of 20%. Considering the increasing public health concern over the double burden of malnutrition in Vietnam, the objective of the present paper is to examine predictors of overweight/obesity among Vietnamese women of reproductive age and to quantify micronutrient deficiencies among Vietnamese women by weight status (underweight, healthy weight, overweight/obese) from the recent 2010 nationwide micronutrient data.

## Methods

### Study design and sampling

The sample size for this study was estimated to evaluate the prevalence of micronutrient deficiency among women of reproductive age with a hypothesis of 50% prevalence, a precision of 5.0% and an expected design effect of 2.0. Anticipating an estimated 17% refusal or absence of women in the selected households, 840 women per stratum (urban/rural) were required [12]. Consequently, a total of 56 urban and 56 rural clusters of 15 households were selected and 1526 women of reproductive age were surveyed.Non-pregnant women of reproductive age (15–49 years) with no severe or chronic illness or current acute infection (e.g. fever, diarrhea, acute respiratory infection) were included in the survey after obtaining informed consent.

### Anthropometry and body composition

The women were invited to come to the Commune Health Center in the early morning. Women were weighed without shoes or sandals and wearing light clothes using a balance with a precision of 0.1 kg (Body Composition Monitor Scale Tanita BC-543, Tokyo, Japan). Height was measured using a wooden height board with a precision of 0.1 cm (UNICEF supply). The percentage of body fat and water and the bone mineral mass were measured with the same balance using Bioelectric Impedance Analysis taking gender, age, height and weight into account.

Anthropometric data were entered into EpiData (version 6.0). Body mass index (BMI) was calculated and women were classified using the BMI cut-off points for Asian populations: underweight (BMI<18.5 kg/m^2^), healthy weight (BMI 18.5–22.9 kg/m^2^), overweight (BMI 23.0–27.49 kg/m^2^), and obese (BMI≥27.5 kg/m^2^) [13].

### Blood sampling and analysis

After anthropometry data collection (between 0700 and 0900 hours), a non-fasting venous blood sample (6 ml)was withdrawn by venipuncture into trace-element free heparinized sampling tubes (Vacuette, Greiner Bio One, Austria) by licensed medical technicians. Blood samples were stored in the dark in a cool box and transported within 4 hours of collection to the Provincial Medical Center (PMC) laboratory for hemoglobin determination. Hemoglobin concentration (Hb) was then measured immediately according to the manufacturer’s instructions using a HemoCue device (Hemocue 301, Ängelholm, Sweden) and control material provided by Hemocue (HemoCue - HemoTrol, Keplerlaan, Netherland). Thereafter, plasma was obtained by centrifugation at 3000 g for 10 min at 4°C. Plasma was aliquoted into 200 µL pre-labelled Eppendorf tubes and kept frozen at −20°C at the Provincial Medical Center before being sent, within two weeks, on dry ice to the National Institute of Nutrition (NIN) in Hanoi, where samples were stored at −70°C until analysis.

Plasma concentrations of ferritin, retinol, zinc, and C-reactive protein (CRP) were determined at the NIN Micronutrient Laboratory. Plasma ferritin was measured by ELISA using commercial kits that included reference samples (Ramco Laboratories Inc, Houston, Texas). The accuracy was tested with Bio-Rad liquicheck control (Bio-Rad Laboratories, USA). Plasma retinol concentration was determined by reverse-phase HPLC (LC-10 ADVP, Shimadzu, Japan) according to the method of the International Vitamin A Consultative Group in a dimly lighted room [14]. Zinc was analysed using a flame atomic absorption spectrophotometer (GBC, Avanta+) using trace element-free procedures and powder free gloves (Latex Surgical Glove), and results were verified using reference materials (Liquicheck, Bio-Rad Laboratories, USA). A control for potential contamination of material used for blood sampling and blood processing by external zinc was carried out by analysing zinc content of at least 20 sets of materials (needle, syringe, vacutainers and eppendorf tubes) where blood was replaced by bi-distilled water. CRP, an acute phase protein, was used to quantify the acute phase response as a marker of inflammation and infection. Plasma CRP was measured by ELISA using a commercial kit from GenWay (Biotech, Inc., San Diego, California), with reference materials included in each assay (Liquicheck, Bio-Rad Laboratories, USA).

Aliquots of plasma were sent to the Swiss Vitamin Institute (Epalinges, Switzerland) for analysis of folate and vitamin B_12_ using a microbiological assay. Extracted folates were diluted with basal medium containing all required growth nutrients except folate[15]. The growth response of *Lactobacillus casei*, subspecies rhamnosus (ATCC 7469) to extracted folates was compared to the growth response to calibration solutions with known concentrations. Vitamin B_12_ was measured for a randomly selected sub-sample of 505 women. Vitamin B_12_ was analyzed turbidimetrically using *Lactobacillus plantarum* (ATCC 8014) as the test organism [16]–[18]. The growth response of *Lactobacillus plantarum* is proportional to the quantity of vitamin B_12_ in the sample[19]. The within-assay variability for plasma ferritin, retinol, folate, zinc, vitamin B_12_, and CRP was <6%.

Anemia was defined according to WHO standards as hemoglobin below 120 g/l for women of reproductive age [20]. Iron deficiency was defined as plasma ferritin <15 µg/L for women [20] using a correction factor of 0.65 for ferritin concentration from subjects with sub-clinical inflammation as indicated by increased concentrations of CRP (>5 mg/l) [21]. Sub-clinical vitamin A deficiency was defined using WHO cut-offs of plasma retinol <0.7 µmol/l [20], while marginal status was defined as plasma retinol between 0.7 and 1.05 µmol/l [22]. Plasma retinol concentrations were corrected where sub-clinical infection existed [23]. Zinc deficiency was defined using the International Zinc Nutrition Consultative Group (IZiNCG) cut off of zinc <10.1 µmol/l for women [24]. Vitamin B_12_ deficiency was defined as plasma vitamin B_12_ values below 148 pmol/l; vitamin B_12_ status was considered marginal when the plasma concentration was between 148–220 pmol/l [25]. Folate deficiency and marginal status was defined as plasma folate concentrations <13.4 nmol/l [25].

### Socioeconomic status

The Demographic and Health Survey (DHS) Wealth Index was used to divide the surveyed households into five socio-economic quintiles: the “extreme poor” (category 1), the “poor” (category 2), the “intermediate” (categories 3 and 4), and the “wealthy” (category 5). The Wealth Index was constructed from recorded data on household assets such as tables, chairs, refrigerator, air conditioners and beds and also from housing conditions (materials of house floor, house roof, main wall) and facilities (energy for cooking, electricity and latrines) [26].

### Ethical approval

The Scientific Committees of the National Institute of Nutrition (NIN) (Hanoi, Vietnam) and the Ministry of Health (Hanoi, Vietnam) reviewed and approved the study protocol. All women were informed verbally and in writing about the aims and procedures of the study, and written informed consent was obtained from all women before enrolment.

### Statistical analysis

Data entry, including quality checks was performed with Excel 2007. Data management and analysis were performed with SAS software version 9.3 (SAS, V9.3; SAS institute, Cary, NC). All analyses accounted for the cluster sampling design using appropriate SAS survey procedures. Categorical variables are expressed as percentages and standard error percentages (surveyfreq procedure). Bivariate relationships between categorical variables were assessed using the Rao-Scott Chi-Square Test. Bivariate and multivariate relationships between age category, socioeconomic category, ethnic group, urban/rural location, region, education level, and micronutrient status and BMI category (dependent variable) were assessed using ordinal logistic regression or multinomial logistic regression in cases where the proportional odds assumption was not met (surveylogistic procedure). Continuous variables are expressed as means and standard error of the mean (surveymeans procedure). Associations between maternal BMI category, age, and socio economic status and continuous dependent variables (plasma micronutrient concentrations, percent body fat, bone mass) were assessed using linear regression models (surveyreg procedure). Plasma micronutrient concentrations were transformed to natural logarithms as necessary to meet the assumptions of normality of residuals and homogeneity of variance.

## Results

Using the BMI cut-offs for Asian populations, 20.5% of the Vietnamese women were underweight (UW, **Table 1**), while 20.0% were overweight including only 2.4% of obese women. For the remainder of this paper, overweight and obese women (OW) are combined into one category (overweight/obese; BMI≥23 kg/m^2^) with an average BMI in this category of 25±2 kg/m^2^. BMI was highly correlated to maternal body fat (β = 0.91; P<0.001), but less strong to Wealth Index (beta = –0.07 p<0.001) and age (beta = 0.03; p = 0.012).

**Table 1:** Prevalence of underweight, healthy weight, overweight and obesity among Vietnamese women.

Maternal BMI (kg/m^2^) Category	n*	Prevalence (%)	Standard Error ofPrevalence (SEP)	95% CI for prevalence**
Severe underweight (BMI<16)	30	2.0	0.4	1.3–2.7
Moderate underweight (BMI 16–16.9)	58	3.9	0.5	2.9–4.8
Mild underweight (BMI 17–18.49)	220	14.7	1.1	12.5–16.8
Healthy weight (BMI 18.5–22.9)	893	59.5	1.4	56.7–62.3
Overweight (BMI 23.0–27.4)	264	17.6	1.1	15.5–19.7
Obese (BMI≥27.5)	36	2.4	0.4	1.5–3.2

*24 of 1526 women did not have any anthropometric measurements.

**: CI: confidence interval.

Average maternal body fat significantly increased with BMI category (p<0.001), with percent body fat averages (mean±SEM) of 21.0%±0.2% for UW (95% CI: 20.6–21.4), 27.7%±0.1% for healthy weight women (HW, 95% CI: 27.5–28.0) and 35.3%±0.2% for OW (95% CI: 34.8–35.8). Maternal bone density also significantly (p<0.001) increased with BMI category, with bone density averages of 1.66 g/cm^2^±0.02 for UW (95% CI: 1.63–1.70), 1.88 g/cm^2^±0.01 for HW (95% CI: 1.86–1.90) and 2.13g/cm^2^±0.02 for OW (95% CI: 2.09–2.16).

**Figure 1:**
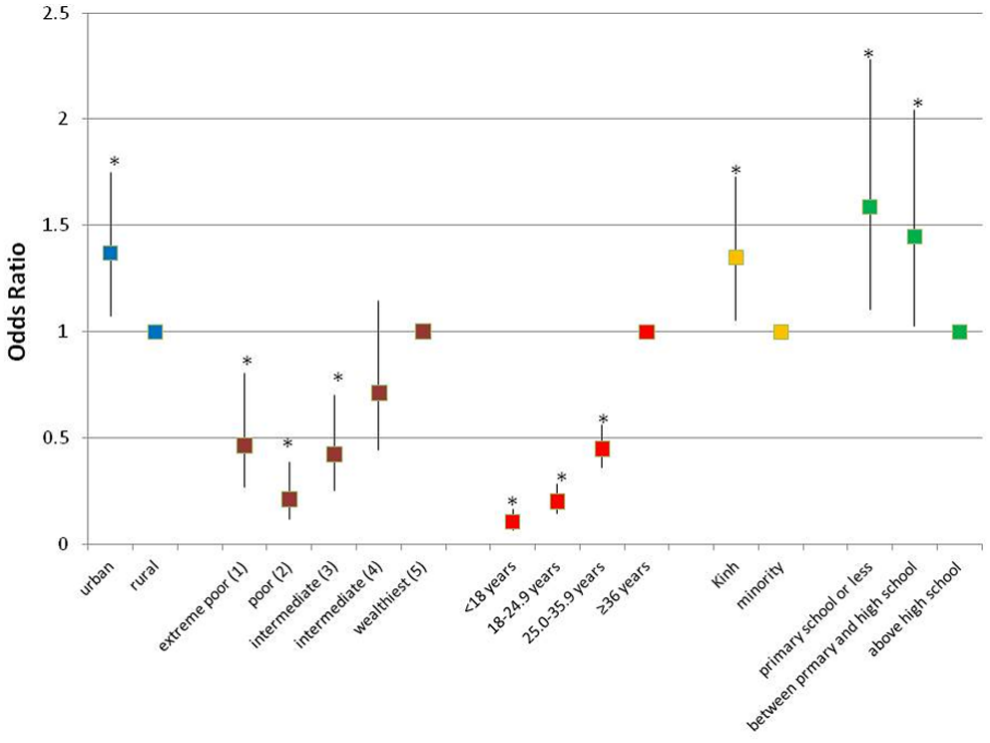
Unadjusted odds ratios for overweight/obesity for Vietnamese women of reproductive age.

**Figure 2:**
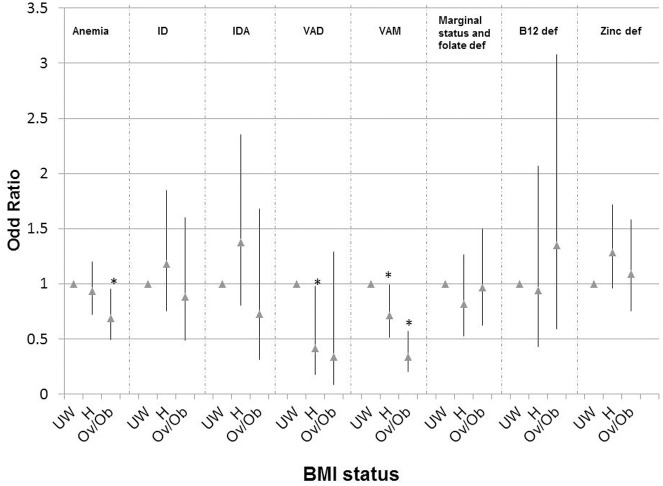
Unadjusted odds ratios for overweight/obesity for Vietnamese women of reproductive age. Note: UW: underweight women; H: healthy women; Ov/Ob: overweight or obese women; ID: iron deficiency; IDA: Iron deficiency anemia; VAD: vitamin A deficiency; VAM: vitamin A marginal status; def: deficiency.

In bivariate analyses, weight status was significantly associated (**Table 2**) with age, daily energy intake, socioeconomic category, ethnicity, urban/rural location, and region. The prevalence of overweight/obesity steadily increased with older age category with 64% of the 300 OW being 36.0 year old or older. OW had significantly higher energy intakes with half of them consuming more than 1900 kcal/day (7955 kJ/day) while ∼60% of UW consumed less than 1660 kcal/day (6950 kJ/day). There was a 55% to 89% reduction in the unadjusted odds of overweight/obesity among women in younger age groups compared to women ≥36 years old (**Figure 1**). Women from the highest socioeconomic group (group 5) clearly had the lowest prevalence of underweight (13.3%) and the highest prevalence of OW. There was a 54% to 79% decrease in the unadjusted odds of overweight/obesity among women in the lowest three socioeconomic groups compared to women in group 5 (**Figure 1**). The prevalence of overweight/obesity was significantly higher among the Kinh ethnic group compared to minority groups and among urban dwellers compared to rural dwellers (22.2% vs. 17.9%), with an 35% increase in the unadjusted odds of overweight/obesity among Kinh vs. minority and urban vs. rural populations (**Figure 1**). The prevalence of overweight/obesity differed significantly by region of the country, ranging from 11.4% in the Central North to an alarming 56.4% in the Central south (**Table 2**). Higher educated women tended to have a lower prevalence of overweight/obesity than less educated women, with an 45 to 59% increased unadjusted odds of overweight/obesity among women with a primary school or high school level education compared to the higher educated women (**Figure 1**). Controlling only for age, % body fat was significantly associated to concentrations of hemoglobin (beta = 0.12; p<0.001), ferritin (beta = 0.05; p = 0.049), retinol (beta = 0.12; p<0.001) and zinc (beta = 0.08; p = 0.02) Given the high correlation between % body fat and BMI, it was not surprising there were significant bivariate relationships between BMI category and hemoglobin, ferritin and retinol concentrations (Table 3), which were significantly lower for UW and HW compared to OW. These relationships persisted after controlling for maternal age and socio economic status, with the exception of ferritin that was no longer significant (p>0.05). A significant association was also observed between BMI category and marginal vitamin A status (p<0.0001). There was, a 66% decrease in the odds of marginal vitamin A status among OW compared to UW (**Figure 2**). Among OW, one of ten women had anemia and iron deficiency whereas 15% had vitamin B_12_ deficiency, 25% marginal/deficient folate status and more than 60% zinc deficiency (Table 3). Consequently, among the women surveyed nation-wide, the intra-individual double burden of malnutrition (overweight/obesity and micronutrient deficiency) was observed among 2.0% (95% CI: 1.3–2.7) for OW-anemia, 2.3% (95% CI: 1.4–3.1) OW-Iron deficiency, 3.0% (95% CI: 1.7–4.3) for OW-Vitamin B_12_ deficiency, 12.2% (95% CI: 10.7–13.7) for OW-Zinc deficiency and 5.2% (95% CI: 4.0–6.3) for OW-marginal/deficient folate status.

**Table 2:** Bivariate relationships betweenage, socioeconomic status, ethnicity, location (rural/urban), region and the prevalence of underweight, normal weight, overweight and obesity among Vietnamese women.

	BMI Category (kg/m^2^)												p*
	Underweight (BMI<18.5)				Healthy Weight (BMI 18.5–22.9)				Overweight/Obese (BMI≥23.0)				
Category	n	Prevalence (%)	SEP	95% CI	n	Prevalence (%)	SEP	95% CI	n	Prevalence (%)	SEP	95% CI	
***Maternal Age (years)*** **													<0.0001
<18	64	49.6	4.7	40.3–58.9	60	46.5	4.4	37.8–55.2	5	3.8	1.7	0.5–7.2	
18–24.9	77	32.1	3	26.1–38.1	150	62.5	3.2	56.2–68.8	13	5.4	1.4	2.7–8.1	
25.0–34.9	102	18.9	1.8	15.2–22.5	348	64.3	2.2	60.0–68.6	91	16.8	1.4	14.1–19.6	
≥36.0	65	11	1.5	8.1–13.9	335	56.7	2.1	52.5–60.9	191	32.3	2.2	28.0–36.6	
***Maternal daily Energy intake (kcal)*** **													<0.0001
<1600 kcal/day	46	59.7	6.7	46.3–73.1	29	37.7	7.1	23.5–51.8	2	2.6	1.7	0.0–6.1	
1600–1899 kcal/day	93	19.8	1.9	15.9–23.7	328	69.8	2.1	65.6–73.9	49	10.4	1.4	7.6–13.2	
≥1900 kcal/day	12	4.1	1.1	2.0–6.2	136	46.6	2.9	40.8–52.3	144	49.3	3	43.4–55.3	
***Socioeconomic category*** **													<0.0001
1	58	23.5	3.5	16.6–30.3	142	57.3	3.8	50.0–65.0	47	19	2.7	13.7–24.3	
2	62	27.3	3.2	20.9–33.7	142	62.6	3.5	55.7–69.5	23	10.1	2	6.1–14.2	
3	64	23.4	3.1	17.4–29.5	162	59.3	3.6	52.2–66.5	47	17.2	2.4	12.5–21.9	
4	66	21	2.1	16.8–25.2	166	52.9	2.7	47.5–58.3	82	26.1	2.5	21.2–31.0	
5	58	13.3	1.7	10.16.6	277	63.5	1.9	59.8–67.2	101	23.2	1.8	19.5–26.8	
***Ethnicity*** **													0.05
Kinh	220	19.1	1.4	16.2–22.0	693	60.2	1.6	57.0–63.3	239	20.7	1.3	18.1–23.3	
Minority groups	82	25.9	2.5	20.9–30.9	178	56.2	3.2	49.8–62.5	57	18	2.3	13.5–22.4	
***Education level*** **													0.09
1	103	18.7	1.7	15.2–22.1	330	59.9	1.9	56.0–63.8	118	21.4	1.9	17.6–25.2	
2	153	20.4	1.7	17.0–23.8	446	59.5	1.8	55.9–63.0	151	20.1	1.5	17.2–23.1	
3	47	28.3	3.4	21.5–35.1	92	55.4	3.7	48.0–62.8	27	16.3	3	10.3–22.3	
**Location** **													0.02
Rural	180	23.3	2	19.3–27.3	454	58.8	2.2	54.5–63.1	138	17.9	1.6	14.8–21.0	
Urban	128	17.6	1.5	14.6–20.5	439	60.2	1.7	56.8–63.6	162	22.2	1.6	19.0–25.5	
**Region** **													<0.0001
Central Highland	19	13.2	2.5	8.2–18.1	101	70.1	3.6	63.1–77.2	24	16.7	2.6	11.6–21.7	
Central North	30	38	7.3	23.5–52.4	40	50.6	6.1	38.5–62.8	9	11.4	3.4	4.7–18.1	
Central South	40	18.7	3.3	12.1–25.3	118	55.1	2.5	50.1–60.1	56	56.4	3.5	49.5–63.2	
Mekong River Delta	27	16.4	3.1	10.2–22.6	93	56.4	3.5	49.5–63.2	45	27.3	2.8	21.7–32.8	
North East	60	25.5	2.8	20.1–31.0	145	61.7	4	53.8–69.6	30	12.8	2.2	8.4–17.1	
North West	13	13.5	3.5	6.6–20.5	61	63.5	7.1	49.5–77.6	22	22.9	3.9	15.1–30.7	
Red River Delta	20	16.5	2.9	10.7–22.3	78	64.5	3.1	58.2–70.7	23	19	2.6	13.9–24.1	
South East	99	22.1	2.3	17.5–26.8	257	57.5	2.5	52.6–62.4	91	20.4	2.3	15.7–25.0	

Note: *Based on bivariate analysis; Rao-Scott Chi-Square Test.

**Number of women with missing data varies by variable: age (n = 25), region (n = 25),location (n = 25), socioeconomic data (n = 29), ethnicity (n = 57), education level (n = 59).

Education level. 1: primary school degree or less; 2 more than primary school but not over high school degree; 3: over high school degree.

**Table 3:** Bivariate relationships between micronutrient status and the prevalence of underweight, normal weight, and overweight/obesity among Vietnamese women.

	Maternal BMI Category (kg/m^2^)													p*
	Underweight (BMI<18.5)				Healthy Weight (BMI 18.5–22.9)Increasing but acceptable risk					Overweight/Obese (BMI≥23.0)Increased/High Risk				
Category	n	Prevalence (%)/mean	SEP/SEM***	95% CI	n	Prevalence (%)/mean	SEP/SEM***	95% CI		n	Prevalence (%)/mean	SEP/SEM***	95% CI	
***Plasma concentration***														
Hemoglobin in g/l	308	130.8	0.07	129.9–132.1	892	131.2	0.05		130.3–132.1	300	132.9	0.07	131.6–134.2	0.04
Ferritin in µg/l	307	70.48	6.89	56.81–84.15	892	73.98	3.18		67.58–80.20	298	91.01	6.56	80–104.03	0.03
serum retinol in µmol/l	298	1.32	0.03	1.27–1.37	861	1.47	0.03		1.42–1.52	290	1.61	0.05	1.51–1.70	<0.0001
Folate in nmol/l	286	18.4	0.6	17.3–19.6	809	18.5	0.4		17.6–19.3	266	18.0	0.5	16.9–19.1	0.543
Vitamin B_12_ in pmol/l	95	580.1	40.4	499.1–661.1	300	660.2	27.1		606.4–713.9	100	577.3	46.5	484.3–670.2	0.106
Zinc in µmol/l	307	9.6	0.2	9.2–9.9	891	9.4	0.1		9.2–9.7	298	9.7	0.2	9.3–10.0	0.097
***Anemia*** **														0.62
No	273	88.6	1.8	85.0–92.3	785	88.0	1.3		85.4–90.6	270	90.0	1.7	86.5–93.5	
Yes	35	11.4	1.8	7.7–15.0	107	12.0	1.3		9.4–14.6	30	10.0	1.7	6.5–13.5	
***Iron deficiency***														
No	268	87.3	2.2	83.0–91.6	761	85.3	1.4		82.5–88.1	264	88.6	2.2	84.3–92.9	0.39
Yes	39	12.7	2.2	8.4–17.0	131	14.7	1.4		11.9–17.5	34	11.4	2.2	7.1–15.7	
***Iron deficiency anemia***														0.09
No	293	95.4	1.1	93.3–97.6	836	93.8	0.9		92.1–95.6	288	96.6	1.0	94.6–98.7	
Yes	14	4.6	1.1	2.4–6.7	55	6.2	0.9		4.4–7.9	10	3.4	1.0	1.3–5.4	
***Vitamin A deficiency*** **														0.08
No	289	97.0	1	94.9–99.1	850	98.7	0.4		98.0–99.4	287	99.0	0.6	97.8–100.0	
Yes	9	3.0	1	1.0–5.1	11	1.3	0.4		0.6–2.0	3	1.0	0.6	0.0–2.2	
Marginal vitamin A status^**^														<0.0001
No	242	81.2	2.2	76.8–85.6	739	85.8	1.3		83.3–88.4	269	92.8	1.5	89.7–95.8	
Yes	56	18.8	2.2	14.4–23.2	122	14.2	1.3		11.6–16.7	21	7.2	1.5	4.2–10.3	
***Zinc deficiency*** **														0.18
No	126	41.0	4	33.2–48.9	313	35.1	2.9		29.4–40.9	116	38.9	3.8	31.4–46.4	
Yes	181	59.0	4	51.1–66.8	578	64.9	2.9		59.1–70.6	182	61.1	3.8	53.6–68.6	
***Vitamin B_12_ deficiency*** **														0.58
No	84	88.4	3.6	81.2–95.6	267	89.0	2.2		84.7–93.3	85	85.0	3.3	78.5–91.5	
Yes	11	11.6	3.6	4.4–18.8	33	11.0	2.2		6.7–15.3	15	15.0	3.3	8.5–21.5	
***Folate deficiency and marginal status*** **														0.71
No	211	71.5	2.9	65.9–77.2	623	72.1	2.0		68.2–76.1	213	74.2	2.9	68.5–80.0	
Yes	84	28.5	2.9	22.8–34.1	241	27.9	2.0		23.9–31.8	74	25.8	2.9	20.0–31.5	

Note: *: Based on linear regression (with variables log transformed as needed to meet the assumptions of normality of residuals and homogeneity of variance) for mean and the Rao-Scott Chi-Square Test for prevalence.

**Number of women with missing data differ by micronutrient: folate (n = 80), vitamin B_12_ (n = 1031), zinc (n = 30), vitamin A (n = 77), and anemia (n = 28).

***SEP/SEM: Standard Error of the Mean/Standard Error of the Prevalence.

## Discussion

Earlier, we reported a double burden of malnutrition at the country level among Vietnamese women of reproductive age, with an overall prevalence of both underweight and overweight/obesity of approximately 20% [12]. The present study demonstrates that a significant proportion of Vietnamese women of reproductive age experience the double burden of malnutrition at intra-individual level, with overweight/obesity and micronutrient deficiency being present in the same individual. Especially zinc deficiency was prevalent, both in overweight/obese and normal weight women, with >60% of the women affected. Only the prevalence of vitamin A deficiency was lower in OW women, as compared to UW women, whereas the prevalence of deficiency or marginal status of iron, folate or vitamin B_12_ did not differ between underweight, normal weight and overweight/obese women.

The poor status of vitamin B_12_ (15%) and folic acid (26%) in these overweight/obese Vietnamese women, even though not different in prevalence to normal weight women, is reason for concern as deficiency of either vitamin B_12_ or folic acid can lead to an increase in plasma homocysteine levels [27], [28], which is a powerful risk factor for cardiovascular disease [29]. Combined with the elevated BMI, these women are even at a higher risk for cardiovascular disease. Therefore national strategies to improve vitamin B_12_ and folic acid status in the general population are needed.

Ferritin concentrations (corrected for inflammation [21]), increased significantly with BMI as reported earlier from finding in the USA [30]. There is some controversy around iron status in obese subjects. Some authors have suggested that higher hepcidin levels in obesity may contribute to lower iron absorption and hence to iron deficiency in obesity [31]. Also, obesity is characterized by higher rates of inflammation, which will lead to higher concentrations of plasma ferritin, thereby causing underestimation of iron deficiency in subjects with obesity [32]. In the present study, we corrected ferritin concentrations for inflammation, using the factors derived by Thurnham et al [21] that have been confirmed relevant for obesity recently [32]. Even after correction for inflammation, higher % body fat (and hence higher BMI) is still associated with higher ferritin concentrations, showing that in this population, overweight/obesity is not a risk factor for iron deficiency, and even associated with better iron status. This is in accordance with another study showing that overweight Mexican women had similar anemia and iron status (measured with transferrin saturation) compared to normal weight women [33]. In the same study however, obese women had lower iron status. In the present study, most of the women were overweight (average BMI: 25±2 kg/m2), based on cut-offs adapted for Asian population [13], with only 0.6% having a BMI over 30 kg/m2 [12], the obesity cut-off used in studies in Western populations, perhaps explaining this difference with the study from Mexico.

In contrast to the water-soluble vitamins, there were clear differences for vitamin A status between underweight, normal weight and overweight Vietnamese women, with vitamin A status being highest in overweight/obese women, with significant higher plasma retinol concentrations and an Odd Ratio of 0.38 for marginal vitamin A status among overweight/obese women compared to underweight women. Another study from Mexico [34] among 580 women of reproductive age did not find any difference in serum vitamin A concentration among BMI categories. However, the authors did not correct retinol concentrations for inflammation. Retinol concentrations are known to decrease with inflammation [23]. Hence, vitamin A status in these obese Mexican women might have been underestimated.

The higher iron and especially retinol status among Vietnamese overweight/obese population could be explained by a higher dietary diversity. We showed that overweight/obese participants had much higher energy intake than underweight women and higher socio-economic level. This energy increase might have consequently induced higher micronutrient intakes as a previous study in Vietnam shows that non-poor Vietnamese households experienced an significant increases in calorie intake and dietary diversity during the period 1997/1998–2004 [35] while poor households did not experience such an increase. Furthermore, in Vietnam, the consumption of vitamin A has been positively correlated to the household expenditure [36]. As both iron and vitamin A deficiency are associated with anemia [37], [38], the better iron and retinol status in overweight women may also explain the higher haemoglobin concentration observed in overweight/obese Vietnamese women.

In conclusion, in the present study there was a positive association between % body fat on one side, and hemoglobin and plasma retinol, zinc and ferritin concentrations on the other side, even after correcting retinol and ferritin concentrations for inflammation. The apparent discrepancy between studies on iron and vitamin A status in overweight/obese subjects is likely due to the effect of inflammation on indicators of iron and vitamin A status [39]. No correction for inflammation seems to lead to an overestimation of iron status, and an underestimation of vitamin A status in overweight/obese subjects. Another factor which could explain the discrepancies is the prevalence of overweight and obese subjects in the study population, with different effects being reported between overweight and obese subjects. When using global cut-offs for obesity, in contrast to Asian cut-offs, the prevalence of obesity was very low in the present study (0.6% of the women [12]). The higher iron and vitamin A status in our population is most likely due to a higher food intake which has not lead to a better status of water-soluble vitamins (folate and B_12_). Knowing the close link of folate and B_12_ deficiency and the risk for cardiovascular disease, it is essential for Vietnam to actively monitor the progress of overweight and obesity to limit health consequences for the population as well as the economic burden for the country [40]. It may also be worthwhile to monitor waist circumference, as excess of central adiposity is strongly associated with the increase of non-communicable disease prevalence [41]. But BMI should be also kept as an indicator as other research says that no strong evidence supports replacing BMI in clinical or public health practice with other adiposity measures [42].

## Conclusions

This large, cross-sectional survey demonstrates that micronutrient deficiencies were an issue across the weight spectrum among women in Vietnam. Thus it is important to not only focus on underweight women but also on overweight/obese women for micronutrient deficiency prevention programs, even in higher socioeconomic strata. Improving micronutrient status of overweight/obese women is particularly important because micronutrient deficiencies may increase chronic disease risk. The findings of this study highlight the importance of targeting interventions to improve micronutrient status for whole populations, and not restrict those to populations selected on basis of specific nutritional status only (such as low BMI).

Given the demonstrated health and economic costs of micronutrient deficiencies and obesity, heart disease and diabetes, it is urgent that national interventions and policies address both the trend towards increasing overweight/obesity and the on-going problem of micronutrient deficiencies in Vietnam.

## Supporting Information

Data S1 **Dataset.**
(SAV)
